# A new discovery of the bioluminescent terrestrial snail genus *Phuphania* (Gastropoda: Dyakiidae)

**DOI:** 10.1038/s41598-023-42364-y

**Published:** 2023-09-13

**Authors:** Arthit Pholyotha, Daichi Yano, Gaku Mizuno, Chirasak Sutcharit, Piyoros Tongkerd, Yuichi Oba, Somsak Panha

**Affiliations:** 1 Animal Systematics Research Unit, Department of Biology, Faculty of Science, Chulalongkorn University, Bangkok, 10330 Thailand; 2 Department of Environmental Biology, Chubu University, Kasugai, 487‑8501 Japan; 3 Academy of Science, The Royal Society of Thailand, Bangkok, 10300 Thailand

**Keywords:** Ecology, Evolution, Zoology

## Abstract

The mysterious world of the bioluminescent molluscs in terrestrial ecosystems is mesmerizing, but *Quantula striata* was previously the only terrestrial mollusc known to be luminescent. Here, we document the new discovery of bioluminescence in four land snails, namely *Phuphania crossei*, *P. globosa*, *P. carinata*, and *P. costata.* Our observations establish clearly that these four species of *Phuphania* produce a continuous greenish light from the light-emitting cells located within the mantle and the foot, and that its bright luminescence is intracellular and is not due to any luminous secretion. Although both *Quantula* and *Phuphania* can produce a green light, the luminescence patterns are different. The luminescence displayed by *Quantula* is rhythmical blinking or flashing, while *Phuphania* glows continuously. In addition, the bioluminescence in *Q. weinkauffiana* is confirmed, which is similar to that in the related species, *Q. striata*.

## Introduction

Bioluminescence is the phenomenon of light emission by living organisms through a chemical reaction in their bodies^1, 2^. Although bioluminescence is relatively rare in nature, examples of species with bioluminescence are found in diverse taxa ranging from bacteria and protists to squid and fishes^1, 2^. In contrast to bioluminescence, biofluorescence is a phenomenon by which living organisms exhibit visible fluorescence when irradiated by shorter-wavelength light^3, 4^. The fluorescence sometimes can be used to identify the anatomical position of the bioluminescent organs because the components of bioluminescence are generally fluorescent^5–8^.

In Mollusca, bioluminescence is an unevenly distributed trait, where luminous species have only been found in three of the eight taxonomic classes: Cephalopoda, Gastropoda, and Bivalvia. The most prominent of the luminous molluscs are the Cephalopoda, which contain a large number of luminous genera^1, 9^. On the other hand, luminous molluscs, excluding cephalopods, are comparatively rare. Three genera of the Bivalvia (*Cucullaea*, *Gastrochaena*, and *Pholas*) and nine genera of the Gastropoda (*Kalinga*, *Phylliroe*, *Kaloplocamus*, *Plocamopherus*, *Melanella*, *Hinea* (= *Angiola*), *Planaxis*, *Latia*, and *Quantula*) are also known to be luminous^1, 9^. All luminous molluscs presently known are oceanic, except the freshwater limpet *Latia* from New Zealand and one member of the terrestrial snail genus *Quantula* Baker, 1941 from mainland Southeast Asia^1, 9^.

*Quantula* is one of the diverse genera in the family Dyakiidae Gude & Woodward, 1921 and is currently comprised of eight species^10^. The phenomenon of bioluminescence in this genus has been known since the discovery of the first luminous species, *Q. striata* (Gray, 1834), by Haneda in 1942 in Singapore^6, 11^. Twenty-six years later, Bassot and Martoja^12^ reported the luminescence of a land snail from Cambodia; this species was first identified as *Q. weinkauffiana* (Crosse & Fischer), but was subsequently changed to *Q. striata* in an addendum to the paper^12^. As a consequence, *Q. striata* is currently recognized as the only terrestrial mollusc known to be luminescent in the world^7, 13^. This luminous snail produces flashes of light similar to those of nocturnal fireflies, and its luminescent organ is located within the pedal gland complex in the anterior head-foot^7, 8, 11^.

Prior to this study, there had been no additional records of new luminous terrestrial snails. Here, we report our recent discovery of four luminous species in Thailand belonging to the genus *Phuphania* Tumpeesuwan et al., 2007, which is closely related to the genus *Quantula*^14, 15^. We also observed the luminescence of* Q. weinkauffiana* during a recent field survey in Thailand and established its authentic classification, making it the second luminescent *Quantula* species. In the present study, we aim to describe the bioluminescent characteristics of these species. We also describe the biofluorescence and ultrastructure of the tissues to identify the position and morphology of the luminous organs.

## Results

### Observations of luminous phenomena

**Figure 1:**
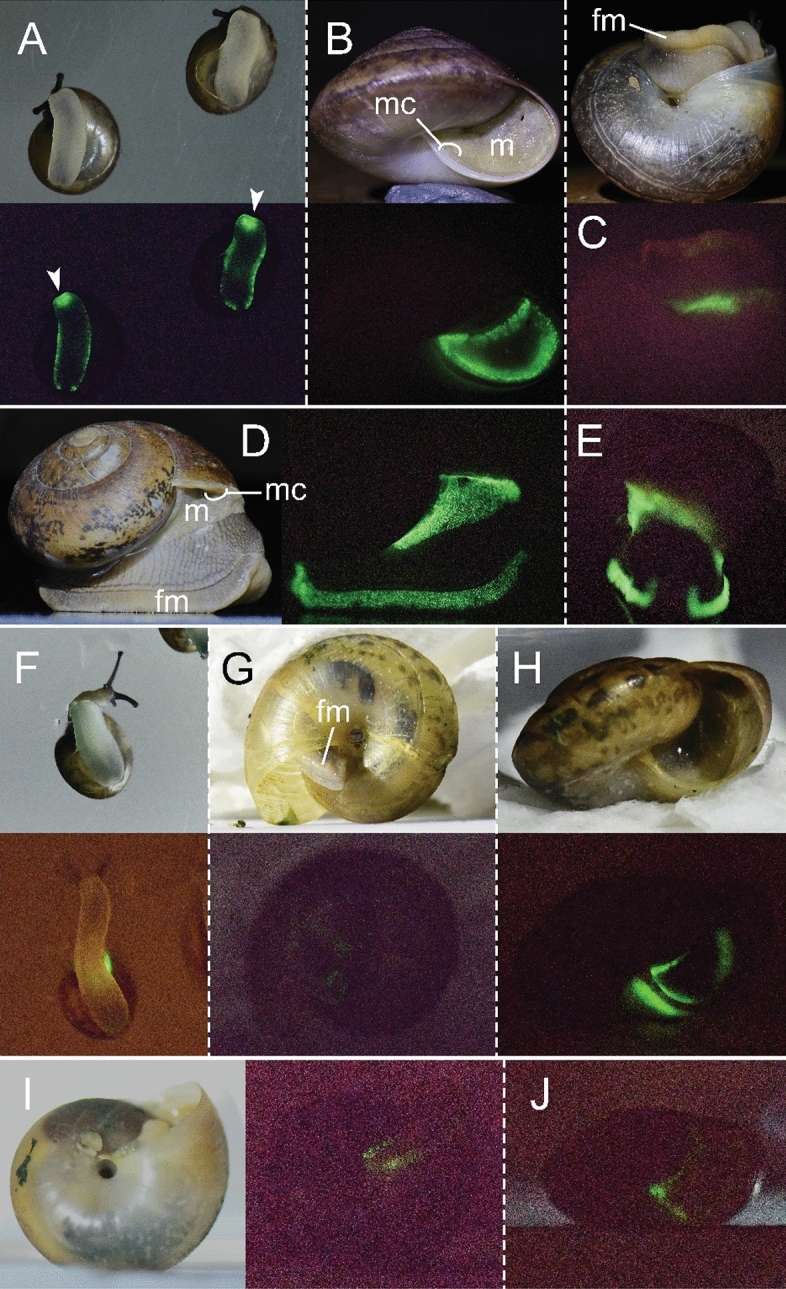
Bioluminescence of the terrestrial snail genus *Phuphania.* (**A**) Juveniles of *P. crossei* and (**B**, **C**) adults of *P. crossei* in natural light (above) and in the dark (below); arrows indicate the light at the anterior foot. (**D**, **E**) Adult of *P. globosa*; (**D**) lateral view in natural light (left) and in the dark (right) and (**E**) frontal view in the dark. (**F**–**H**) Juveniles of *P. costata* in natural light (above) and in the dark (below). (**I, J**) Juvenile of *P. carinata*; (**I**) ventral view in natural light (left) and in the dark (right) and (**J**) lateral view in the dark. Images of living snails are not to scale. Abbreviations: fm = foot margin; m = mantle; mc = mantle collar.

Luminescence from the snails was investigated in four *Phuphania* species [*P. crossei* (Pfeiffer, 1862), *P. globosa* Tumpeesuwan et al., 2007, *P. carinata* Kongim & Panha, 2013, and *P. costata* Tumpeesuwan & Tumpeesuwan, 2014]. The luminous phenomena of these four *Phuphania* species can be observed in both daytime and night-time under natural conditions without stimulation. They emitted a continuous green light (Fig. 1; Supplementary Video 1) that was weak but visible to the human eye in the dark. When compared by eye to the light of nocturnal fireflies, such as the common Thai firefly *Pteroptyx malaccae*, the light of *Phuphania* as well as *Quantula* is considerably weaker in intensity. The light appears either as a weak diffuse glow over the anterior foot or as a weak luminescence from many small points in the foot margin and the mantle. No luminous secretion was found from any of the four species of *Phuphania*. The position of luminescence spontaneously produced by *P. crossei* is a little different from that for *P. globosa* and *P. costata*. In juveniles (n = 14) and adults (n = 9) of *P. crossei*, the luminescence is emitted by the luminous cells located on the anterior foot, behind the mouth (white arrows in Fig. 1A); on the edge of the mantle or mantle collar (Fig. 1B,C); and on the foot margin (Fig. 1A,C). The light-emitting cells on the foot margin of adult *P. crossei* are less concentrated than in juveniles. The light organ at the anterior foot could not be found in *P. globosa* and *P. costata.* In adult *P. globosa* (n = 1), the light-emitting cells are found over the entire mantle (Fig. 1D,E) and on the foot margin (Fig. 1D,E). In juvenile *P. costata* (n = 5), the luminescence is emitted by the light-emitting cells situated mostly on the mantle collar (Fig. 1F,H) and on the foot margin (Fig. 1G). Bioluminescence in *P. carinata* (n = 1) was only observed at the mantle, with the light-emitting cells being situated mostly on the edge of the mantle (Fig. 1I,J).

### Fluorescence spectra of the luminous *Phuphania crossei* and *P. globosa*

**Figure 2:**
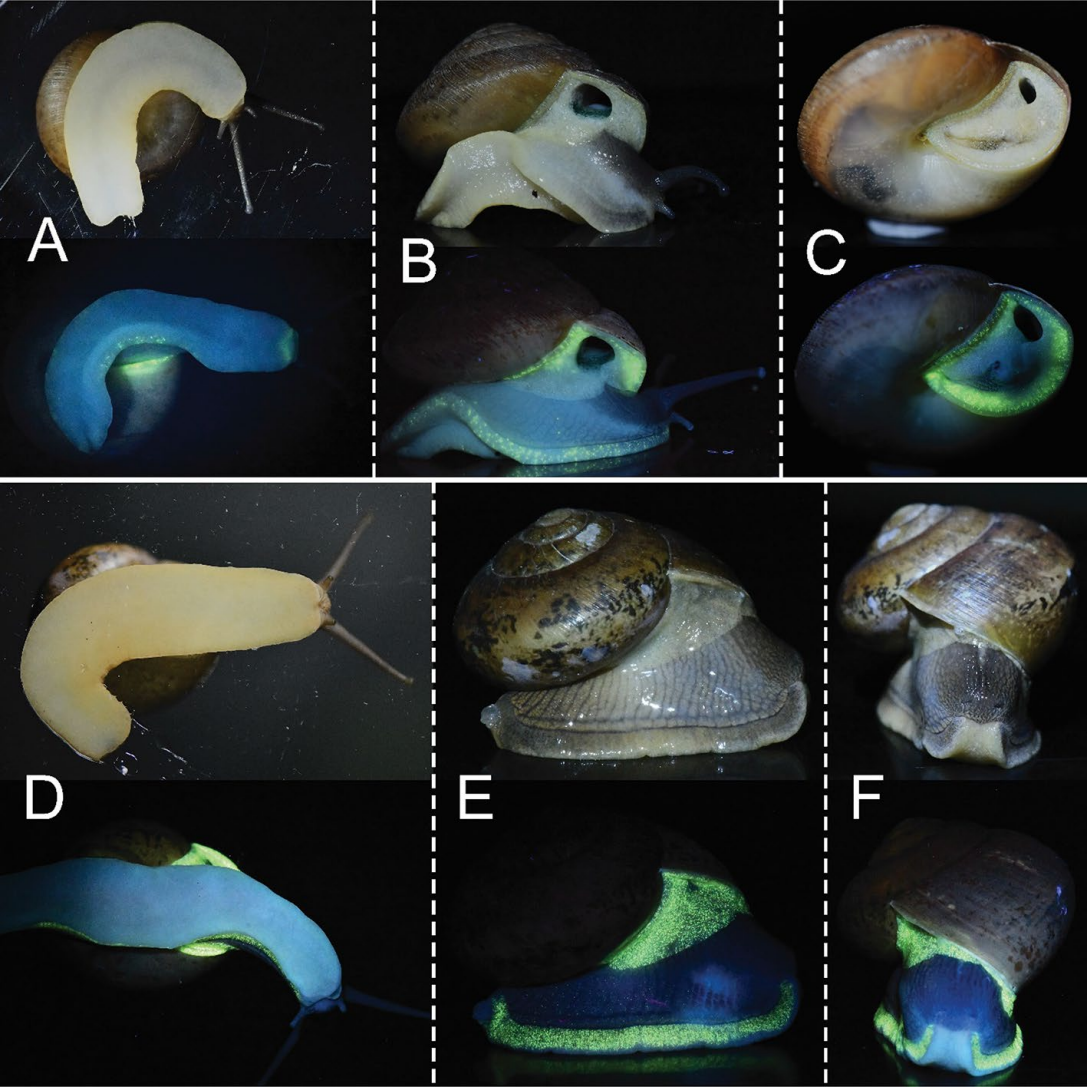
Biofluorescence of the terrestrial snail genus *Phuphania.* (**A–C**) Adults of *P. crossei* under UV light (365 nm) (above) and under natural light (below). (**D–F**) Adults of *P. globosa* under UV illumination (above) and under natural light (below). Images of living snails are not to scale.

**Figure 3:**
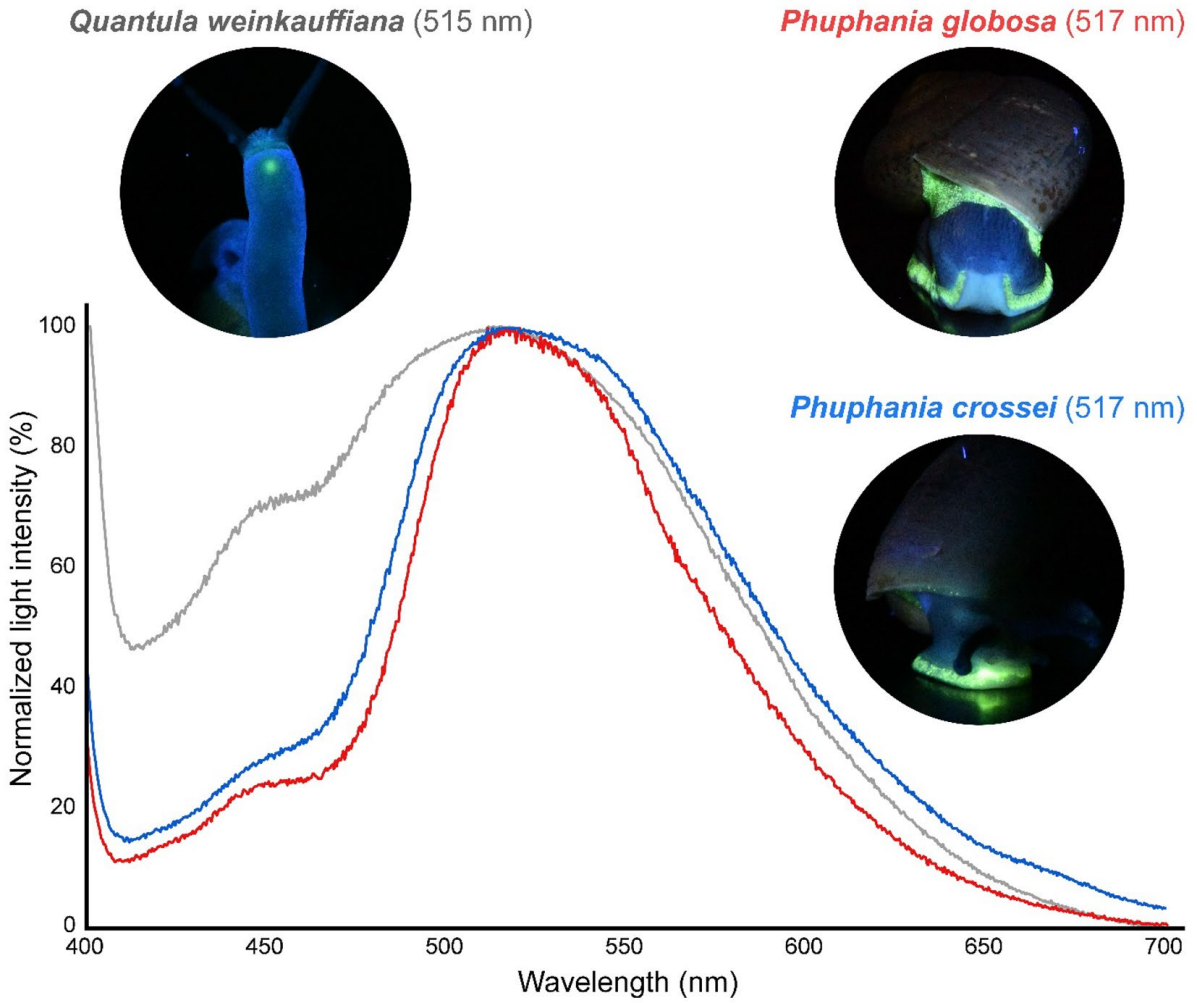
Biofluorescent emission spectra in terrestrial snails. The maximum emission wavelengths of the biofluorescent light emitted by *Phuphania crossei* (maximum emission at 517 nm), *P. globosa* (maximum emission at 517 nm), and *Quantula weinkauffiana* (maximum emission at 515 nm) are green when illuminated with UV light (365 nm).

Under 365-nm ultraviolet (UVA) radiation, the soft body of both *P. crossei* and *P. globosa* showed a strong greenish fluorescence (Fig. 2A–F), which can be seen well by the naked human eye. Areas of biofluorescence in *P. crossei* (Fig. 2A–C) and *P. globosa* (Fig. 2D–F) were concentrated around the foot and mantle, which correspond to the locations of the photogenic tissues (Fig. 1). The maximum emission of in vivo fluorescence in both *P. crossei* and *P. globosa* was at 517 nm (Fig. 3); however, the fluorescence excitation and emission spectra of *P. carinata* and *P. costata* have not yet been investigated.

### Histology of the luminous *Phuphania crossei*

**Figure 4:**
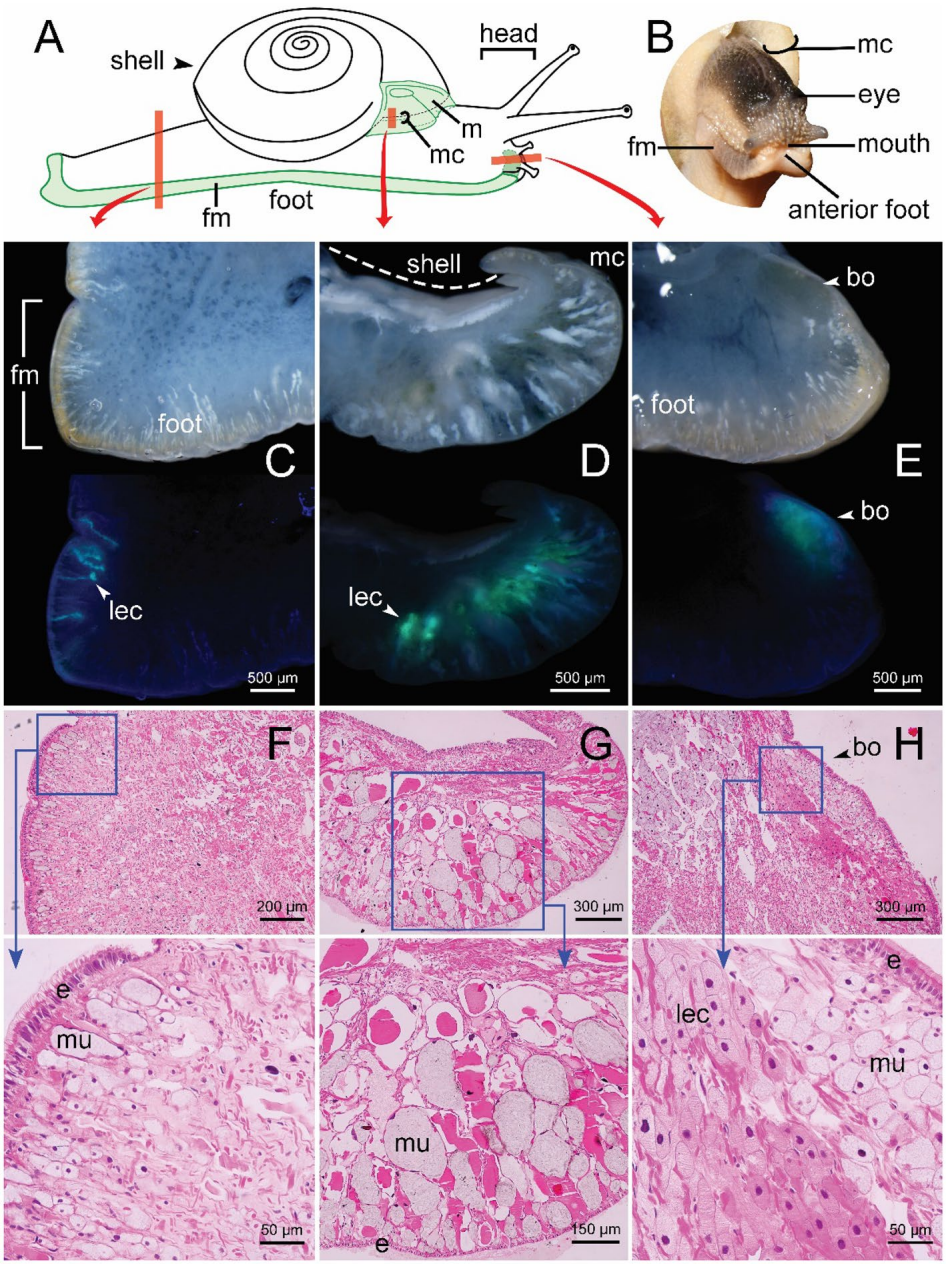
Histology of the luminous *Phuphania crossei.* (**A**) Red boxes indicating schematic position of histological samples. (**B**) Frontal view of living snail showing anterior foot. (**C**–**E**) Green fluorescence from thick sections of the fresh tissues illuminated with UV light (365 nm); (**C**) transversal section of foot margin; (**D**) transversal section of mantle collar; (**E**) longitudinal section of anterior foot. (**F–H**) Histological sections, stained with hematoxylin and eosin; (**F**) transversal section of foot margin; (**G**) transversal section of mantle collar; (**H**) longitudinal section of anterior foot. Abbreviations: bo = bioluminescent organ; e = epidermis; fm = foot margin; lec = light-emitting cells; m = mantle; mc = mantle collar; mu = mucous cells.

Since the luminescence of *P. crossei* is localized in a limited area, we examined histological sections of the mantle, foot margin, and anterior foot to establish the localization of their bioluminescent tissues (Fig. 4A,B). Before preparing histological sections, thick sections of the fresh foot (Fig. 4C), mantle collar (Fig. 4D), and anterior foot (Fig. 4E) were examined. We detected transparent cells exhibiting a strong greenish fluorescence under UV light (365 nm).

For the histological study, we could not identify the light-emitting cells in the photogenic layers of the foot margin or the edge of the mantle. The histology of the foot and mantle of this species has not been studied previously but resembles in most respects the typical land snail structure^16, 17^. The foot margin is composed of a simple columnar ciliated epithelium, below which are connective tissue cells and mucous glands (Fig. 4F). In the luminous area of the mantle collar, under the thin epithelium, there appears a large number of mucous glands and many cellular masses (Fig. 4G), which very likely contain the bioluminescent tissues of this part. At the anterior foot, we could determine the photogenic organ (Fig. 4H). The organ runs along the anterior foot from the left to the right side, and is covered by a layer of mucous cells below a simple columnar epithelium. Mucous cells (pale violet in colour) lining the interior are many-layered, spherical, and slightly larger in size than the photogenic cells. Beneath the mucous cells, there is a layer of photogenic cells within which a few muscle fibres are found intermingled. Two types of the photogenic cells are present, many-layered and oval, with different staining properties (pale pink or pink in colour).

### COI phylogeny

**Figure 5:**
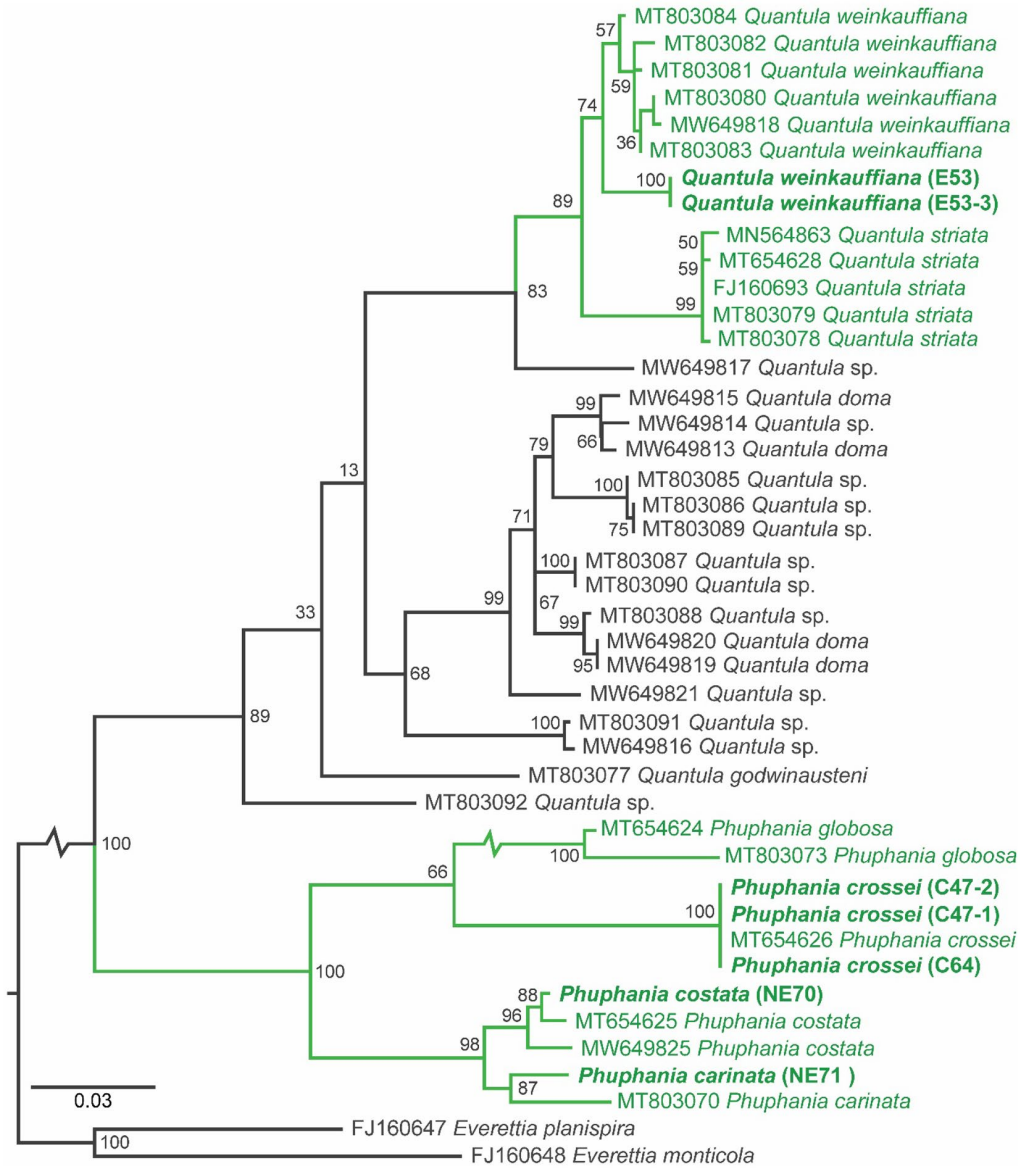
Maximum likelihood tree of the dyakiid genera *Phuphania* and *Quantula* based on the COI gene dataset. Numbers on nodes indicate the ML bootstrap values (%). Species names in bold indicate the new COI sequences provided by this study.

The final sequence dataset contained 11 sequences of *Phuphania* and 30 sequences of *Quantula* together with two sequences of *Everettia* used to root the tree (Supplementary Table S1). The obtained maximum likelihood (ML) phylogenetic tree (Fig. 5) recovered all the species of *Phuphania* and *Quantula* as a well-supported clade. Even though the relationships among *Quantula* taxa were poorly resolved, a clade of *Q. weinkauffiana* was always retrieved as a sister group of *Q. striata* with high support (Fig. 5). While the relationships among the four species of *Phuphania* taxa were resolved, a clade of *P. crossei* + *P. globosa* appears as a sister group of *P. carinata* + *P. costata* with high support (Fig. 5).

## Discussion

**Figure 6:**
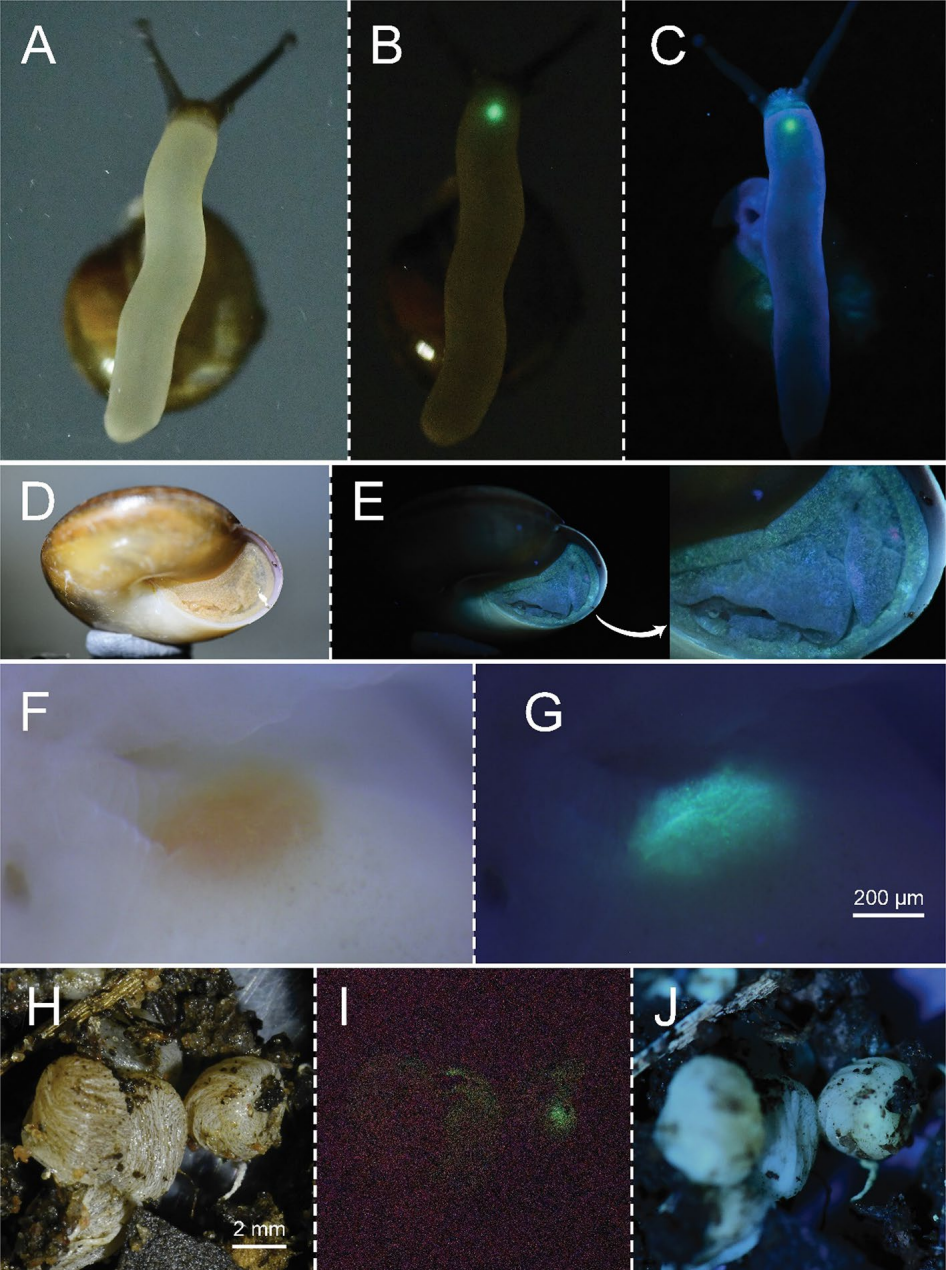
Bioluminescence, fluorescence, and histology of the luminous *Quantula weinkauffiana*. (**A**) Ventral view in natural light. (**B**) Ventral view in the dark. (**C**) Ventral view under UV light (365 nm). (**D**) Lateral view in natural light. (**E**) Lateral view under UV light. (**F**) Bioluminescent organ preserved in formalin in natural light. (**G**) Bioluminescent organ under UV light. (**H**–**J**) Eggs (**J**) in natural light, (**I**) in the dark, (**J**) under UV light.

Bioluminescence of a land snail was first discovered in *Quantula striata* by field observation of specimens in Singapore^11^. In this study, we found a bioluminescent land snail in Thailand, and based on the morphological analysis, it was determined to be *Q. weinkauffiana*. This luminous species produced discrete flashes (< 6 s) of greenish light (Fig. 6A,B), sometimes single-peaked and sometimes multiple-peaked (Supplementary Video 2), similar to that of *Q. striata*^18, 19^. The correct classification of the species as *Q. weinkauffiana* was supported by molecular analysis (Fig. 5), confirming the presence of two luminous species in the genus *Quantula*. Currently, it is known that *Q. striata* is native to Singapore and Peninsular Malaysia, and has been introduced to Borneo^20^ and Fiji^21^; there is no evidence of this species in eastern or north-eastern Thailand, Laos, or Cambodia based on the recent land snail literature from this region^22–24^. On the other hand, *Q. weinkauffiana* is widely distributed in eastern and north-eastern Thailand^24^, Laos^22^, and Cambodia^23^, but there are no records of its distribution in the Malay Peninsula. Based on the separated distributions and morphological similarity of *Q. striata* and *Q. weinkauffiana*, we suspect that the luminescent specimens observed in Cambodia by Bassot and Martoja in 1968 were *Q. weinkauffiana*, as they first concluded.

In this study, we discovered the greenish glow of another dyakiid genus, namely *Phuphania*. The luminescence was confirmed in all species of the genus *Phuphania* (*P. crossei*, *P. globosa*, *P. carinata*, and *P. costata*). Continuous or long-lasting luminescence was observed in the mantle and foot, which is in contrast to those of the *Quantula* species, which emit a discontinuous (blinking) luminescence from a spot on the anterior foot. We also confirmed the lack of bioluminescence and biofluorescence in another dyakiid species *Pseudoplecta bijuga* (Stoliczka, 1873) (Supplementary Fig. S1). Recent molecular studies^15^ showed that the genera *Phuphania* and *Quantula* form a clade together with the genus *Khmerquantula* Pholyotha & Panha, 2021, and that *Pseudoplecta* is located outside of this clade, suggesting the possibility of bioluminescence and biofluorescence in the genus *Khmerquantula*.

*Phuphania* snails employ luminous organs to emit light. The organs are often arranged in species-specific patterns in various locations over the body, including the mantle, the foot margin, and the anterior foot. In *P. globosa*, the luminous cells are scattered over the entire mantle and foot margin. In contrast, the luminous cells of the remaining species of *Phuphania* are restricted to the edge of the mantle. Among these four *Phuphania* species, only *P. crossei* has the light organ on the anterior foot. In the thick tissue sections, the organ showed little difference in colour from the other tissues (preserved in formalin) but had a strong greenish fluorescence under UV light (Fig. 4E). The histological analysis showed that the photogenic layer of the anterior foot consists of two types of photogenic cells showing different staining properties (pale pink and pink). However, at the foot margin and the edge of the mantle, we could only state with certainty what region the photogenic layer was located in, because it was difficult to clearly determine the specific luminous cells when the tissue was stained with hematoxylin and eosin. They are likely to be associated with some of the other cell types in the region, but further histochemical analyses and ultrastructural investigation are needed to clarify this. A reflector layer, chromatophore layer, or lens structures, which are sometimes present in the light organ of various luminous organisms, were not observed in any light organs of *Phuphania* species.

Similarly, the luminescent organ of *Quantula*, called ‘the organ of Haneda’ (Martoja and Bassot^7^) is situated below the mucous fold at the anterior foot, and its luminous cells are concentrated around the middle of the anterior foot, visible as a pale-yellow spot (preserved in formalin; Fig. 6F), and distinctly seen under UV light (Fig. 6C,G). In the organ of Haneda, the photocytes are large cells in which the cytoplasm contains many photocyte granules^7, 8^. Haneda and Tsuji^25^ also reported that the mantle, foot, and even the eggs in *Q. striata* also displayed a weak and diffuse luminescence^25^. The reflector layer, chromatophore layer, and lens structures were not present in the light organ, as in *Q. striata*. In this work, the eggs of *Q. weinkauffiana* (Fig. 6H) were also found to have a greenish glow (Fig. 6I) but without any fluorescence under UV light (Fig. 6J). Although luminescence in the mantle was not observed, very weak and greenish fluorescent dots were visible under UV light (Fig. 6D,E).

In gastropods and bivalves, bioluminescent light is emitted from specialized structures called luminous organs or photophores. These organs contain the light-emitting cells or photogenic cells from which light is generated intracellularly or from which a luminous secretion is synthesized and discharged by the animal^26^. Formation of an extracellular luminescence, where gland cells are filled with the luminous slime to be extruded to the outside of the body by the contraction of muscles, occurs in the luminous gastropods *Plocamopherus* and *Latia*, and in the luminous bivalves *Cucullaea*, *Gastrochaena*, and *Pholas*^9, 26–28^. The luminescence of *Phuphania* is intrinsic and takes place within bioluminescent cells or photocytes (intracellular luminescence) without any bioluminescent secretions, which is similar to that in *Quantula*, *Planaxis*, and *Hinea*^6, 7, 18, 29, 30^.

Our present observations on *Phuphania* showed that the luminous snails can remain aglow continuously for a long time (observed for about 10 min in *P. globose* and *P. crossei*) and that the light emission can be controlled by the animal, because they stop emitting light on some days (observed in juveniles of *P. costata* and both juveniles and adults of *P. crossei*; Supplementary Fig. S2), but the controlling factors and the mechanisms are unknown. In addition, the *Phuphania* snails appeared to conserve their bioluminescent abilities during several weeks after collection, even in hibernation. It is possible that the luminous snails may have a particular metabolic mechanism to maintain luminescence, for example, the luciferin recycling system that is known in the fungal bioluminescent system^31, 32^. Based on the fact that they sometimes glow continuously while in a stationary (non-moving) stage and with no stimulation, we hypothesize that the biological function of the *Phuphania* bioluminescence is to mimic that of other terrestrial organisms that use their luminescence as aposematic displays. For example, some fireflies are distasteful and/or toxic, and the luminescence at their stationary stages of eggs and pupae have been considered to have a role as an aposematism to warn off predators^33^, while one of the possible functions of luminescence in the luminous mushrooms is as a display of their toxicity against fungivorous animals^34^. It is suggestive that the luminescence color of *Phuphania* is green, like firefly eggs and pupae and mushrooms; green is probably the most visible colour to nocturnal predators in terrestrial settings^35^.

Further experiments relating the luminescence in *Phuphania* to its biochemistry have not yet been attempted, and the biochemical control of bioluminescence in the well-known luminous *Quantula* is still poorly understood. In this study, the spectrum of *Q. weinkauffiana* fluorescence had an emission maximum near 515 nm under UV light (Fig. 3), similar to that of *Q. striata*, which showed a maximum wavelength at about 510–520 nm^36, 37^. In previous work on the *Quantula* bioluminescent system, a green fluorescent substance extracted from the light organ (presumed to be the luminescent substance) showed a fluorescence spectrum with a maximum at 515 nm, and is probably different from the luminescent substance in fireflies^19, 36, 37^. Although the *Phuphania* luminescence spectrum could not be measured in this study because of its weak bioluminescent light intensity, the fluorescence spectra have an emission maximum near 517 nm with a small shoulder near 450 nm in both *P. crossei* and *P. globosa*; these are similar to the fluorescence wavelengths in *Q. weinkauffiana* (λmax = 515 nm; Fig. 3) and *Q. striata*^36, 37^. These results suggest that the fluorescent compound in *Phuphania* might be the same or be similar in chemistry to that of *Quantula*. Since the molecular phylogenetic analysis supported the sister-relationship between *Phuphania* and *Quantula*^14, 15^, we expect that these two genera share a common bioluminescence mechanism.

## Methods

### Specimens

Land snail specimens were collected by hand collecting using intensive visual searching by four people per day of each area within different localities in the rainy season in Thailand. Identification of species followed the original descriptions^14, 24, 38–42^ and were also compared with a reference DNA barcode database of the COI gene of each species (see Supplementary Information). The maturity stage of snails (adult and juvenile) was identified based on shell size and genitalia^14, 24, 38–42^. Twenty-three specimens of different ages of the luminous *Phuphania crossei* were collected from Kaeng Khoi District, Saraburi Province. One adult specimen of the luminous *P. globosa* was collected from Phu Phan District, Sakon Nakhon Province. Four juvenile specimens of the luminous *P. costata* were collected from Mueang District, Loei Province. One juvenile specimen of the luminous *P. carinata* was collected from Nong Hin District, Loei Province. For other dyakiid genera, the luminous *Quantula weinkauffiana* was collected from Makham District, Chanthaburi Province and the nonluminous species *Pseudoplecta bijuga* was collected from Mueang District, Yala Province. Some of these snail specimens were maintained in plastic containers at room temperature, and fed with cucumber, carrot, and mushroom. All work with animals was conducted in accordance with the Chulalongkorn University Animal Care and Use Committee (CU-ACUC) under the approval number 2123023.

### DNA extraction, amplification, sequencing, and COI analysis

Details of samples selected for COI analysis are shown in Supplementary Table S1. Genomic DNA was extracted from the foot tissue using a NucleoSpin Tissue kit (Macherey–Nagel, Germany), according to the manufacturer’s instructions. A fragment of the mitochondrial cytochrome c oxidase subunit I (COI) gene was amplified from each specimen by PCR using the universal primer pair LCO1491 and HCO2198^43^. PCR cycling was performed as 94 °C for 1 min, followed by 40 cycles of 98 °C for 10 s, 51 °C for 30 s, and 72 °C for 90 s, and then followed by a final 72 °C for 5 min. The PCR products were then commercially sequenced by Bioneer Co., Korea.

The COI gene sequences from this study, and those of related species and homologous sequences obtained from the GenBank database using genus name and BLASTn searches were aligned using ClustalW, as implemented in the MEGA7 software^44^. The phylogenetic analyses were conducted using maximum likelihood (ML) in the CIPRES Science Gateway^45^. The ML analysis was performed using the GTRCAT model at the default settings of RAxML-HPC2 on XSEDE v.8.2.12^46^ with 1000 bootstrap replicates.

### Luminescence and fluorescence photography

For the luminescence photography, we imaged living specimens in a dark room, with a DSLR camera (Nikon D850) with a Nikon 105 Macro lens (AF-S VR Micro-Nikkor 105 mm f/2.8G IF-ED) using the following settings: ISO speed, 12,800–25,600; F-stop, f/3.5-f/7.1; and exposure time, 10–30 s. The biofluorescence of the snail specimens was captured under UV light (Alonefire SV38, 5W, 365 nm) using a Nikon camera with a Macro lens (ISO speed, 1250–25,600; F-stop, f/10-f/45; and exposure time, 1/8–1/250 s) in a dark room.

## Histology

Adult specimens (n = 3) of *Phuphania crossei* were used. Each snail was anesthetized by cold temperature (4 °C, 10 min). The shell was then removed and the animal was initially fixed in 10% formaldehyde for 5 min. Animals were cut manually at a thickness of ca. 2 to 4 mm and the fresh photogenic tissues were imaged under UV light (Alonefire SV38, 5W, 365 nm) using an Olympus SZX16 stereo microscope with Olympus Cell’D imaging software. These sectioned tissues were then fixed in 10% formaldehyde and stored at room temperature. Subsequently, the samples were dehydrated in a graded series of ethanol, embedded in paraffin, and sectioned at a thickness of 4 µm using a microtome (Leica RM2235). The sections were mounted on slides and stained with Hematoxylin and Eosin stain (H&E). All slides were examined using light microscopy (Olympus CX31) and photographed with a Canon 750D camera attached to the microscope. The light-emitting cells were identified from histological section photographs (40 × magnification) by their location in the organ based on the previous photographs of thick sections of the fresh photogenic tissues examined under the UV light, and by the cellular morphology, according to Haneda^6, 29^, Nicol^27^, Campion^16^, Martoja and Bassot^7^, Daston and Copeland^8^, and Lőw et al.^17^.

### Fluorescence spectroscopy

We used a spectrometer (Ocean Optics Inc, Flame-S) connected via a bifurcated 600 µm fibre optic probe (Ocean Optics Inc., R600-2-VIS–NIR) to measure the spectrum of fluorescence emissions produced by living snails when irradiated under UV light (Alonefire SV38, 5 W, 365 nm) in a dark room. Fluorescence spectra were recorded from three replicates with the software OceanView 1.6.7.

### Video recording

Video recording of the luminescence in *Phuphania crossei* was performed using a Nikon D500 camera and a Micro NIKKOR 60 mm lens (ISO speed, 64,000; F-stop, f/2.8; and exposure time, 1/60 s), and in *Quantula weinkauffiana* using a Nikon D850 camera and a Micro NIKKOR 105 mm lens (ISO speed, 25,600; F-stop, f/2.8; and exposure time, 1/60 s).

### Supplementary Information

Supplementary Information 1.

Supplementary Video 1.

Supplementary Video 2.

## Data Availability

All data generated or analysed during this study are included in this published article and its Supplementary Information file.
