# Supplementary material for: A new discovery of the bioluminescent terrestrial snail genus Phuphania (Gastropoda: Dyakiidae)
Source: Sci Rep. 2023 Sep 13;13:15137. doi: 10.1038/s41598-023-42364-y (PMC10499882; doi:10.1038/s41598-023-42364-y)
Supplement: Supplementary file 1 — Supplementary Information 1. [file 41598_2023_42364_MOESM1_ESM.docx]

**A new discovery of the bioluminescent terrestrial snail genus *Phuphania* (Gastropoda: Dyakiidae)**

Arthit Pholyotha^1^, Daichi Yano^1^, Gaku Mizuno^2^, Chirasak Sutcharit^1^, Piyoros Tongkerd^1^, Yuichi Oba^2^ & Somsak Panha^1,3^

^1^Animal Systematics Research Unit, Department of Biology, Faculty of Science, Chulalongkorn University, Bangkok 10330, Thailand. ^2^Department of Environmental Biology, Chubu University, Kasugai 487‑8501, Japan. ^3^Academy of Science, The Royal Society of Thailand, Bangkok 10300, Thailand.

Corresponding author. Email: somsak.pan@chula.ac.th (S.P.); yoba@isc.chubu.ac.jp (Y.O.)

**Supplementary information**

**Supplementary Table S1.** List of samples shows species names, GenBank accession numbers, and localities. Specimen codes correspond to codes in the molecular phylogenetic tree in Figure 5. Asterisk indicates the type species of its respective genus.

| Species | Code | GenBank accession number | Locality | Reference |
| --- | --- | --- | --- | --- |
| *Phuphania* Tumpeesuwan et al., 2007 |  |  |  |  |
| *Phuphania costata*  Tumpeesuwan & Tumpeesuwan, 2014 | H67 | MT654625 | Pha Phu, Loei, Thailand | Jirapatrasilp et al., 2021 |
| *Phuphania costata*  Tumpeesuwan & Tumpeesuwan, 2014 | PLL | MW649825 | Loei, Thailand | Deeprom et al., 2022 |
| *Phuphania costata*  Tumpeesuwan & Tumpeesuwan, 2014 | NE70 | OR485694 | Mueang, Loei, Thailand | **This study** |
| *Phuphania carinata*  Kongim & Panha, 2013 | H125 | MT803070 | Phu Kiew, Chiayaphum, Thailand | Jirapatrasilp et al., 2021 |
| *Phuphania carinata*  Kongim & Panha, 2013 | NE71 | OR485695 | Nong Hin, Loei, Thailand | This study |
| *Phuphania crossei* (Pfeiffer, 1862) | H5 | MT654626 | Jedkot Waterfall, Saraburi, Thailand | Jirapatrasilp et al., 2021 |
| *Phuphania crossei* (Pfeiffer, 1862) | C47-1 | OR485696 | Kaeng Khoi, Saraburi, Thailand | **This study** |
| *Phuphania crossei* (Pfeiffer, 1862) | C47-2 | OR485697 | Kaeng Khoi, Saraburi, Thailand | **This study** |
| *Phuphania crossei* (Pfeiffer, 1862) | C64 | OR485698 | Kaeng Khoi, Saraburi, Thailand | **This study** |
| *Phuphania globosa*  Tumpeesuwan et al., 2007* | H12 | MT803073 | Wat Tam Nam Pok, Khon Kaen, Thailand | Jirapatrasilp et al., 2021 |
| *Phuphania globosa*  Tumpeesuwan et al., 2007* | H42 | MT654624 | Nam Pung Reservoir, Sakon Nakhon, Thailand | Jirapatrasilp et al., 2021 |
| *Quantula* Baker, 1941 |  |  |  |  |
| *Quantula godwinausteni* (Laidlaw, 1931) | H30 | MT803077 | Wat Khao Sukim, Chanthaburi, Thailand | Jirapatrasilp et al., 2021 |
| *Quantula striata* (Gray, 1834)* | H23 | MT654628 | Singapore Botanic Gardens, Singapore | Jirapatrasilp et al., 2021 |
| *Quantula striata* (Gray, 1834)* | H26 | MT803078 | Singapore Botanic Gardens, Singapore | Jirapatrasilp et al., 2021 |
| *Quantula striata* (Gray, 1834)* | H56 | MT803079 | Pulau Tioman, Pahang, Malaysia | Jirapatrasilp et al., 2021 |
| *Quantula striata* (Gray, 1834)* | - | FJ160693 | Singapore | Liew et al., 2009 |
| *Quantula striata* (Gray, 1834)* | - | MN564863 | Malaysia | Liew et al., 2020 |
| *Quantula weinkauffiana*  (Crosse & Fischer, 1863) | H18 | MT803080 | Khao Panom Sawai, Surin, Thailand | Jirapatrasilp et al., 2021 |
| *Quantula weinkauffiana*  (Crosse & Fischer, 1863) | H52 | MT803081 | Khong Sedon, Salavan, Laos | Jirapatrasilp et al., 2021 |
| *Quantula weinkauffiana*  (Crosse & Fischer, 1863) | H53 | MT803082 | Tad Pha Suam, Champasak, Laos | Jirapatrasilp et al., 2021 |
| *Quantula weinkauffiana*  (Crosse & Fischer, 1863) | CA11 | MT803083 | Phnom Kampong Trach Cave Temple, Kampong Trach, Kampot, Cambodia | Jirapatrasilp et al., 2021 |
| *Quantula weinkauffiana*  (Crosse & Fischer, 1863) | CA13 | MT803084 | Phnom Chhngok Cave, Tuek Chhou, Kampot, Cambodia | Jirapatrasilp et al., 2021 |
| *Quantula weinkauffiana*  (Crosse & Fischer, 1863) | PSP | MW649818 | Khao Panom Sawai, Surin, Thailand | Deeprom et al., 2022 |
| *Quantula weinkauffiana*  (Crosse & Fischer, 1863) | E53-1 | OR485699 | Makham, Chanthaburi, Thailand | This study |
| *Quantula weinkauffiana*  (Crosse & Fischer, 1863) | E53-3 | OR485700 | Makham, Chanthaburi, Thailand | This study |
| *Quantula doma* Deeprom et al., 2022 | PKJ | MW649815 | Phu Jor Kor, Mukdahan, Thailand | Deeprom et al., 2022 |
| *Quantula doma* Deeprom et al., 2022 | PNY | MW649813 | Pha Nam Yoi, Roi Et, Thailand | Deeprom et al., 2022 |
| *Quantula doma* Deeprom et al., 2022 | KSH | MW649819 | Khao Sala, Surin, Thailand | Deeprom et al., 2022 |
| *Quantula doma* Deeprom et al., 2022 | KSL | MW649820 | Khao Sala, Surin, Thailand | Deeprom et al., 2022 |
| *Quantula* sp. | H122 | MT803085 | Nang Rong Waterfall, Nakhon Nayok, Thailand | Jirapatrasilp et al., 2021 |
| *Quantula* sp. | H24 | MT803086 | Wang Ta Krai Waterfall, Nakhon Nayok, Thailand | Jirapatrasilp et al., 2021 |
| *Quantula* sp. | H50 | MT803087 | Khao Ang Rue Nai, Chacheongsao, Thailand | Jirapatrasilp et al., 2021 |
| *Quantula* sp. | H60 | MT803088 | Wat Khao Sala, Surin, Thailand | Jirapatrasilp et al., 2021 |
| *Quantula* sp. | H94 | MT803089 | Wang Ta Krai Waterfall, Nakhon Nayok, Thailand | Jirapatrasilp et al., 2021 |
| *Quantula* sp. | H97 | MT803090 | Khao Ang Rue Nai, Chacheongsao, Thailand | Jirapatrasilp et al., 2021 |
| *Quantula* sp. | H51 | MT803091 | Kam Hom Waterfall, Sakon Nakhon, Thailand | Jirapatrasilp et al., 2021 |
| *Quantula* sp. | H124 | MT803092 | Cuc Phuong, Nho Quan, Ninh Binh, Vietnam | Jirapatrasilp et al., 2021 |
| *Quantula* sp. | QPMM | MW649814 | Roi Et, Thailand | Deeprom et al., 2022 |
| *Quantula* sp. | QUPP | MW649816 | Sakon Nakhon, Thailand | Deeprom et al., 2022 |
| *Quantula* sp. | QSPF | MW649817 | Surat Thani, Thailand | Deeprom et al., 2022 |
| *Quantula* sp. | QPJN | MW649821 | Ubon Ratchathani, Thailand | Deeprom et al., 2022 |
| *Everettia* Godwin-Austen, 1891 |  |  |  |  |
| *Everettia monticola* Liew et al., 2009 | - | FJ160648 | Mount Kinabalu, Sabah, Malaysia | Liew et al., 2009 |
| *Everettia planispira* Liew et al., 2009 | - | FJ160647 | Tawau Hills Park, Sabah, Malaysia | Liew et al., 2009 |

**References**

Deeprom K, Tumpeesuwan C, Tumpeesuwan S (2022) A new species of the genus Quantula Baker, 1941 (Eupulmonata: Dyakiidae) from the southern part of north-eastern Thailand. Raffles Bulletin of Zoology 70: 519–533. https://lkcnhm.nus.edu.sg/wp-content/uploads/sites/10/2022/11/RBZ-2022-0029.pdf

Jirapatrasilp P, Tongkerd P, Jeratthitikul E, Liew T-S, Pholyotha A, Sutcharit C, Panha S (2020) Molecular phylogeny of the limacoid snail family Dyakiidae in Southeast Asia, with the description of a new genus and species. Zoological Journal of the Linnean Society. 193(1): 250–280. https://doi.org/10.1093/zoolinnean/zlaa129

Liew T, Marzuki ME, Schilthuizen M, Chen Y, Vermeulen JJ, Mohd-Azlan J (2020) Molecular phylogenetics and evolutionary history of the endemic land snail genus *Everettia* in northern Borneo. PeerJ 8:e9416. https://doi.org/10.7717/peerj.9416

Liew T-S, Schilthuizen M, Vermeulen JJ (2009) Systematic revision of the genus *Everettia* Godwin-Austen, 1891 (Mollusca: Gastropoda: Dyakiidae) in Sabah, northern Borneo. Zoological Journal of the Linnean Society 157(3): 515–550. https://doi.org/10.1111/j.1096-3642.2009.00526.x

**Supplementary Figure S1.** A non-bioluminescent dyakiid species, *Pseudoplecta bijuga* (Stoliczka, 1873). (A) Lateral view in visible light. (B, C) Lateral view under UV light (365 nm). (D) Ventral view under UV light.


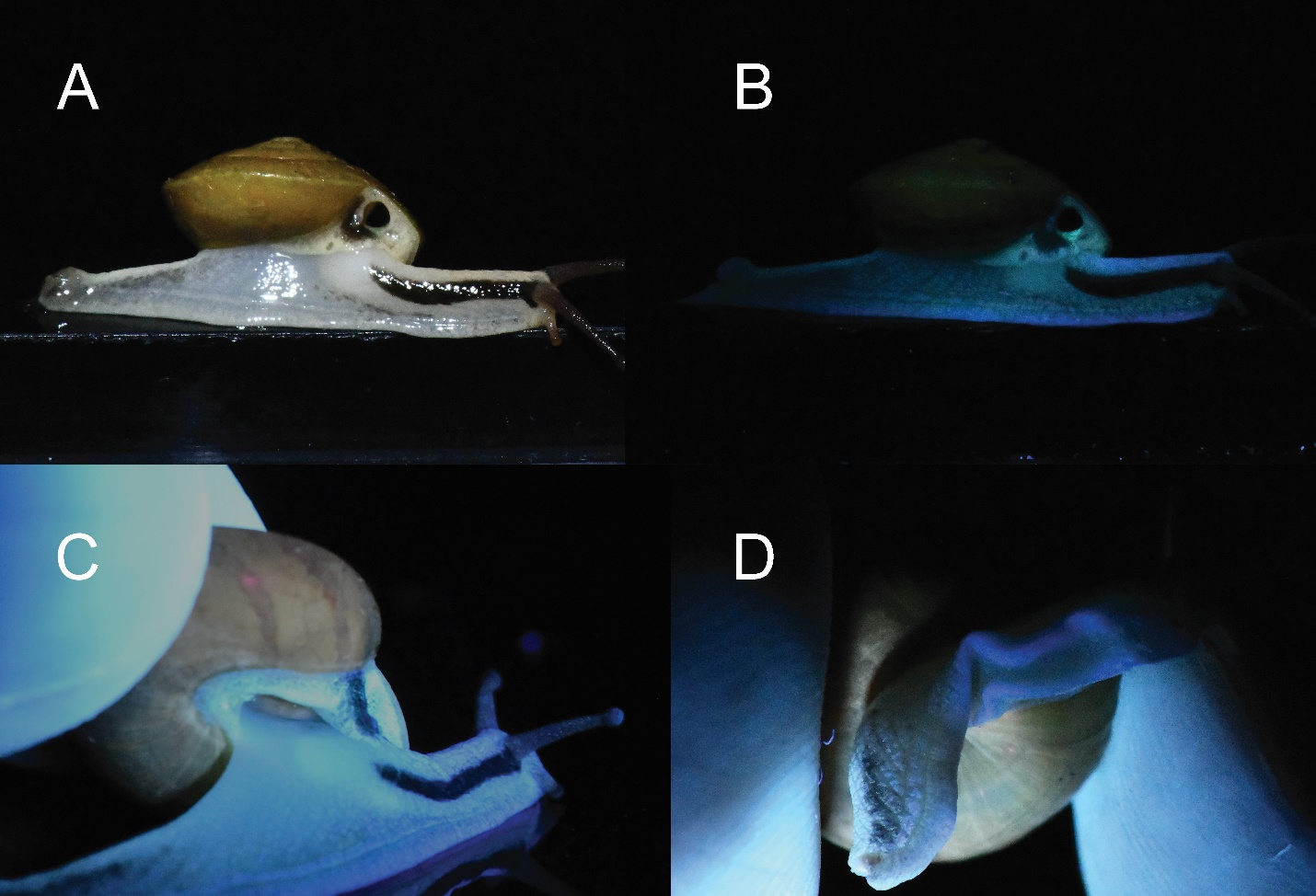


**Supplementary Figure S2.** Bioluminescent activity of the luminous *Phuphania costata* (above) and *P. crossei* (below). Arrows indicate snails with no light emission (pause in bioluminescent activity).

**
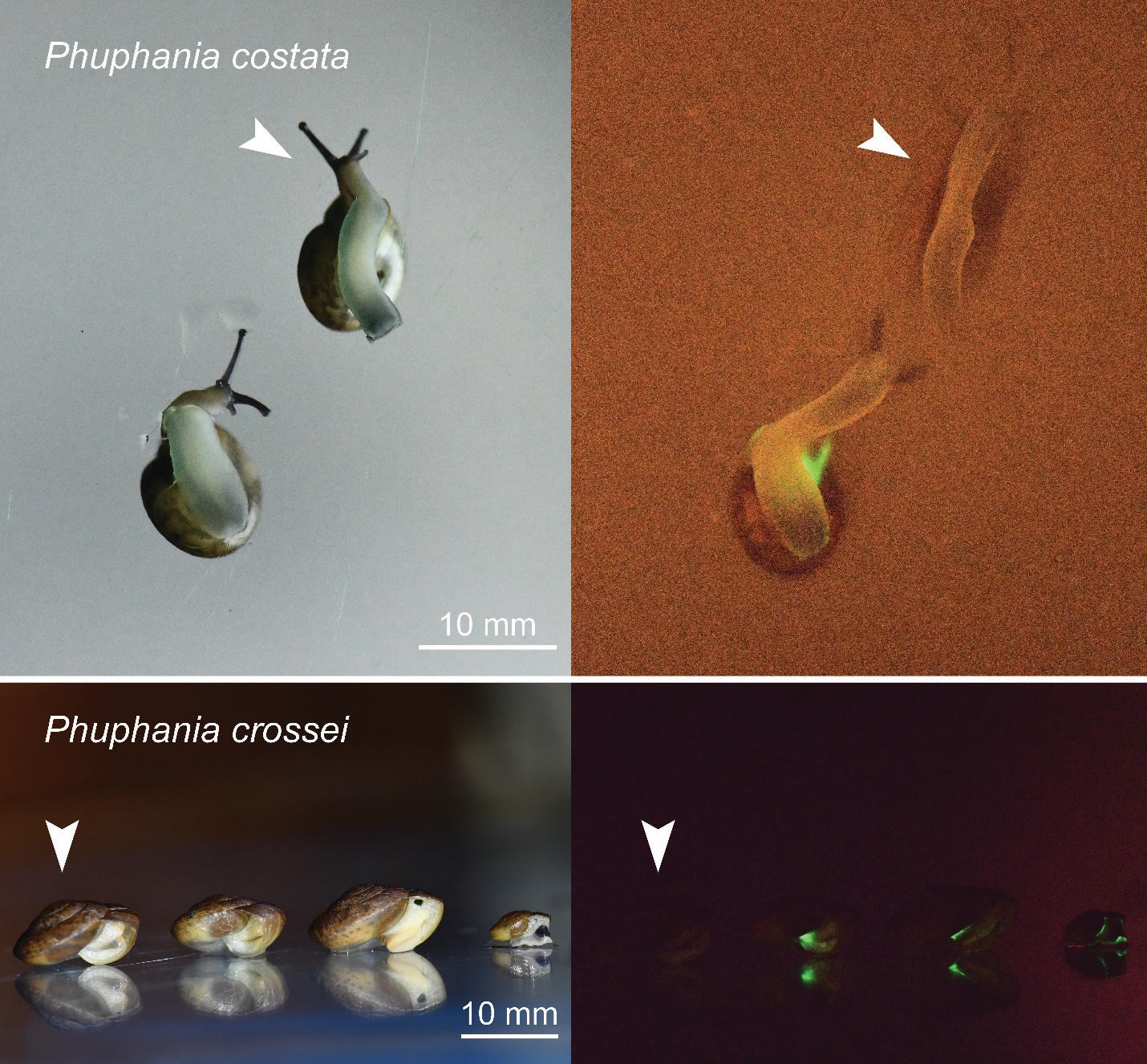
**

**Supplementary Video 1.** Continuous luminescence of *Phuphania crossei*.

**Supplementary Video 2.** Flashing luminescence of *Quantula weinkauffiana*.
